# Comparison Between Browser- and App-Based Versions of a Program for Self-Management of Mild to Moderate Depression: Log Data Analysis of a Convenience Sample

**DOI:** 10.2196/58835

**Published:** 2026-03-20

**Authors:** Katharina Scholze, Caroline Allenhof, Ulrich Hegerl

**Affiliations:** 1 Depression Research Centre of German Foundation for Depression and Suicide Prevention, Leipzig, Germany, 49 034122387420; 2 Depression Research Centre of German Foundation for Depression and Suicide Prevention, Department of Psychiatry, Psychosomatics, and Psychotherapy, Goethe University Frankfurt, Frankfurt, Germany; 3 Goethe University Frankfurt, Department of Psychiatry, Psychosomatic Medicine and Psychotherapy, Goethe University Frankfurt, Frankfurt am Main, Germany

**Keywords:** iCBT, internet-based cognitive behavioral therapy, internet-based treatment, internet- and mobile-based intervention, mobile app, depression, cognitive behavioral therapy, smartphone app, app, usability, data analysis, regression model

## Abstract

**Background:**

Internet-based cognitive behavioral therapy (iCBT) for the treatment of depression has proven to be an effective and accessible option. However, iCBTs tend to have low adherence rates, which may negatively impact their effectiveness. One such iCBT program is the browser-based, proven-effective iFightDepression (iFD) tool. An app-based version of the iFD tool is the iFD app, which was developed to improve usability with a smartphone. The iFD app provides enhanced usability and a more optimized user experience on mobile devices. Additionally, it offers more comfortable interaction with worksheets, reduced text with added videos, and quicker access to the program via the smartphone icon. These improved usability on smartphones and could have an impact on adherence.

**Objective:**

This study investigated (1) whether adherence parameters, that is, the *number of worksheets*, the *number of sessions*, and the *number of workshops*, significantly differed between users of the iFD app and the iFD tool and (2) exploratorily whether symptom reduction (delta Patient Health Questionnaire-9 [PHQ-9] scores) differed after 5 to 9 weeks between the iFD app and the iFD tool, after controlling for covariates.

**Methods:**

We used *t* tests to compare data from 56 participants using the iFD app for 8 weeks with data from 172 participants using the iFD tool in a previous 6-week study. Exploratively, symptom reduction was compared between formats. A multiple regression model was calculated with the delta PHQ-9 score as the dependent variable and format, baseline PHQ-9 score, adherence, current psychotherapy, antidepressants, age, and sex as independent variables.

**Results:**

There was no significant difference between the iFD tool and the iFD app in terms of the *number of sessions per week* (*t*_67.393_=0.920; *P*=.36; corrected *P*=.36), the *number of workshops* (*t*_76.368_=–1.217; *P*=.30; corrected *P*=.36), and the *number of worksheets per week* (*t*_74_=0.984; *P*=.33; corrected *P*=.36). We found no difference in delta PHQ-9 scores between the iFD app and the iFD tool, and baseline PHQ-9 scores were the only significant predictor (*b*=–0.61; *P*<.001).

**Conclusions:**

Despite the improved availability of the app version in daily life, there were no significant differences in the use parameters we analyzed and no differences in symptom reduction. This study provides the first evidence that adherence to iCBT content is comparable for browser- and app-based interventions and that symptom reduction is similar for both formats. However, this study used a convenience sample, and therefore, the results must be interpreted with caution. Notably, in the study of the iFD tool, guidance was provided by the study assistants in a standardized manner. In the pilot study on the iFD app, the amount of guidance varied substantially, as it was provided by the participants’ health care practitioners. These differences in guidance could also have an influence on adherence.

## Introduction

Internet-based cognitive behavioral therapy (iCBT) for the treatment of depression has proven to be an effective and accessible option [1]. However, adherence rates tend to be low [2,3], which may negatively impact effectiveness [4]. One such iCBT is the browser-based, proven-effective [5] iFightDepression (iFD) tool, which has been available since 2014. To improve its usability on smartphones and thus potentially increase adherence, an app-based version of the iFD tool, the iFD app, was developed based on the content of the iFD tool. The iFD tool is accessible via mobile web browsers on smartphones; however, the iFD app provides enhanced usability and a more optimized user experience on mobile devices. Further advantages are that the worksheets can be filled in more conveniently, the amount of text has been reduced in favor of new information videos, and the access is faster through the icon on the smartphone.

Smartphone apps are particularly attractive for health-related applications because, unlike desktop computers or laptops, people tend to carry their smartphones everywhere and to keep them on at all times. In a survey, participants were divided into focus groups and interviewed using open-ended questions about their experiences with apps designed to support a healthy lifestyle [6]. The focus groups were recorded, transcribed, and analyzed to identify frequent topics. One topic that frequently emerged was that participants appreciated health apps on smartphones because they provide a “quick and efficient link to a wealth of information ‘on the go’.”

This raises the question of whether the improved availability and usability of a program on a smartphone has a positive effect on adherence and thus potentially on symptom reduction. Although the dose-response relationship remains a subject of debate due to mixed findings [4], some studies have found no significant impact of adherence on efficacy [7-9], while many others indicate a positive correlation between adherence and treatment effectiveness [10-12]. Nevertheless, it is certainly worthwhile to investigate potential factors that could improve adherence. Outside the field of depression, the evidence appears to support the assumption that a program delivered as a smartphone app may enhance treatment adherence. A study comparing an app to a browser-based and paper diary–based weight loss program showed that the app group had a higher frequency of program use than the browser-based and the paper diary–based program [13]. However, in this case, the content of the 2 programs was similar but not identical. The study was not statistically powered to detect a change in weight loss; however, the results were displayed to give an idea of the effect size. After 6 months, participants in the smartphone group demonstrated a statistically significantly greater reduction in body weight compared with those in the website group. However, no significant difference was observed when comparing the smartphone group to the paper diary group.

To the best of our knowledge, there is only 1 study regarding digital interventions on depression comparing app-based vs browser-based access to identical programs. Watts et al [14] compared the adherence of a mobile app group with a computer group in a program for depression. However, in this study, only the *number of completed lessons* was analyzed and found not to be significantly different based on the format. In this study, we examine various use parameters beyond just the *number of completed lessons*. The frequency of log-ins is also a noteworthy factor, as frequent use may indicate a deeper integration of these programs into daily routines. The number of completed worksheets is also a significant parameter, as a higher completion rate may suggest more active engagement with the content. In combination, the various use parameters provide a more comprehensive insight into how engaged and active users are. This allows us to provide a more informed comparison of adherence between the two formats. The aim of our study is to discover whether the app-based format can achieve higher adherence rates than the browser-based format. The following questions were investigated in this study:

1. Do adherence parameters, that is, the *number of completed worksheets*, the *number of sessions*, and the *number of workshops completed,* significantly differ between users of the iFD app and the iFD tool?
2. Does symptom reduction (delta Patient Health Questionnaire-9 [PHQ-9] score) differ between users of the iFD app and the iFD tool after 5 to 9 weeks, after controlling for covariates such as, age, sex, and adherence (this was an exploratory question)?

## Methods

### Study Design

This study is a secondary analysis of data from 2 different studies. We analyzed data from 56 participants from a pilot study (authors KS, CA, and UH; unpublished data, April 2023) of the newly developed iFD app, along with data from 172 participants from the intervention group of a randomized controlled trial (RCT) using the iFD tool published in 2020 [5]. Participants of the pilot study were instructed to use the iFD app for 8 weeks in addition to treatment as usual (TAU). TAU could include psychotherapy, antidepressants, or watchful waiting. The pilot study was conducted from June 2022 to January 2023. Participants in the intervention group of the RCT (who used the iFD tool) were instructed to use the iFD tool for 6 weeks. All participants were treated by either a physician or a psychotherapist. The RCT was conducted between June 2016 and August 2018.

### Sample and Recruitment

The participants of the pilot study of the iFD app were referred by practitioners experienced with the iFD tool who agreed to test the app with their patients. Participants of the pilot study were aged 18 years or above and received a diagnosis of mild to moderate depression (F32.0, F32.1, F32.9, F33.0, F33.1, and F33.9) or dysthymia (F34.1) from a physician or psychotherapist. In Germany, both licensed psychological psychotherapists and physicians are authorized to diagnose depressive disorders. The practitioners provided the patients’ diagnosis to the study team after the patients had given their written informed consent. At the beginning of the study, participants had a PHQ-9 [15,16] score between 5 and 19.

Recruitment for the RCT of the iFD tool took place throughout Germany via the online channels of the German Foundation of Depression and Suicide Prevention. The participants of the intervention group from the RCT were aged 18 years or above and had a current diagnosis of mild or moderate depressive disorder (F32.0, F32.1, F33.0, and F33.1) or dysthymia (F34.1), according to the Mini International Neuropsychiatric Interview and PHQ-9 (score 5‐14), indicating mild to moderate symptoms.

Sociodemographic data for users of the iFD app and the iFD tool are summarized in Table 1.

**Table 1.** Overview of sociodemographic characteristics.

Variables	Study iFightDepression app (n=56)	Study iFightDepression tool (n=172)	Comparison, *P* value (BH^a^-adjusted *P* value)
Age (years)			.97 (.97)
Mean (SD)	42.79 (14.39)	42.86 (12.36)	
Median (IQR)	44.5 (29.0-57.0)	43.5 (33.8-52.0)	
Female, n (%)	34 (60.7)	137 (79.6)	<.001 (<.001)^b^
Baseline PHQ-9^c^ score, mean (SD)	11.84 (4.12)	9.81 (3.51)	.002 (.002)^d^
Current psychotherapy use, n (%)	40 (71.4)	97 (56.4)	.07 (.08)
Current antidepressant use, n (%)	27 (48.2)	114 (66.3)	<.001 (<.001)^b^

a BH: Benjamini-Hochberg.

b These results are significant (*P*<.001).

c PHQ-9: Patient Health Questionnaire-9.

d This result is significant (*P*<.01).

### Ethical Considerations

The study protocol for the RCT of the iFD tool was reviewed and approved by the Ethics Committee at the Faculty of Medicine, University of Leipzig, on February 11, 2015 (case number 080-15-09032015). The protocol of the pilot study of the iFD app was reviewed and approved by the Ethics Committee at the Faculty of Medicine, Goethe University Frankfurt, on June 11, 2021 (reference number 2021-53). Furthermore, we received confirmation from the Ethics Committee at the Faculty of Medicine, Goethe University Frankfurt, on June 4, 2025 (reference number 2025-2467-Waiver), that no further approval from the ethics committee was required for the secondary analysis. The original informed consent obtained from participants for both studies covered the secondary analysis without the need for additional consent. Data were used in a pseudonymized form for the statistical analysis. The interventions were offered free of charge, and no reimbursement was offered to participants.

### Guidance

Guidance for the iFD app was provided by the practitioners managing the participants’ TAU. The study team asked the practitioners to check in with participants at each appointment during TAU about their experience with the iFD app and to discuss how their work with it was progressing. The practitioners were qualified iFD guides, that is, they had completed a 70-minute webinar and actively used the iFD tool. Few practitioners were using the iFD tool for years and therefore completed the webinar several years ago. Among other things, the iFD workshops were explained in detail in the webinar. Although the webinar referred to the iFD tool, the content of the workshops in the iFD app has not changed. Shortly before the start of the study, the practitioners were given a brief introduction to the iFD app, where differences from the iFD tool were explained. Furthermore, calls at 3 time points were scheduled by the study assistants to collect feedback on the app and discuss any changes in treatment. The study assistants also asked how often and for how many minutes they had spoken to the practitioners about the iFD app to estimate the amount of guidance provided by the practitioner.

The guidance for the iFD tool was provided by the study assistants (psychologists and psychotherapists). The study assistants guided the participants in 5 telephone calls, which were supervised by a senior psychiatrist who was involved in the development of the iFD tool. All study assistants were qualified through the standard webinar process and used a guide for the calls based on the webinar content. The focus of the guideline calls was to motivate the participants rather than discuss the content. The study assistant then noted the duration of the phone calls to determine the amount of guidance.

### Measures

The PHQ-9 completion was mandatory at first log-in in both studies and could be completed again at any time during the intervention. Once a week, patients were asked to complete the questionnaire to monitor their symptoms. The instructions in the iFD tool were adjusted so that the measurement now referred to the period starting from the last time the PHQ-9 was completed. In the iFD app, the original instruction—referring to the past 2 weeks—was retained. The sum scores of the baseline PHQ-9 and the last PHQ-9 completed within the time span of 5 to 9 weeks after registration were used in the current analysis. This time span was chosen based on the time span of the 2 studies (6-week RCT iFD tool and 8-week pilot study iFD app, with a buffer of 1 week).

Anonymized log files were used to examine user behavior. These log files contained time-stamped logs of all activity within the iFD tool or app. From these, composite measures of use were created. We determined the *number of sessions per week*, *the number of workshops completed* (at least 70.0% of the material was seen or read), and *the number of completed worksheets per week* to measure and compare adherence.

### Statistical Analyses

For the comparison of the parameters of adherence between the iFD tool and the iFD app, we used the Welch 2-sample *t* test, chosen for its robustness with unequal variances [17]. *P* values were corrected using the Benjamini-Hochberg method [18].

To compare symptom reduction, a multiple regression analysis was performed with *age*, sex, *baseline PHQ-9*, *format* (app vs browser), *workshops completed*, *sessions per week*, *completed worksheets per week*, *amount of guidance* (min), *current psychotherapy* (yes or no), and *current antidepressants* (yes or no) as predictor variables for the delta PHQ-9. Delta PHQ-9 refers to the mean difference between PHQ-9 scores at registration and the last PHQ-9 filled 5 to 9 weeks after registration. It was used as a measure of symptom change. Negative values indicate a reduction in symptoms, while positive values reflect an increase. Only participants who completed the PHQ-9 questionnaire at least once within 5 to 9 weeks were included in the analysis.

The assumptions of multiple regression were assessed and tested. Normality of the residuals was evaluated using graphical methods, specifically a histogram and a Q-Q plot. The histogram displayed a bell-shaped curve characteristic of a Gaussian distribution, indicating approximate normality. In the Q-Q plot, the majority of data points aligned closely with the reference line, with several lying directly on it. However, the Shapiro-Wilk test indicated a significant deviation from normality (*P*=.01). Given the known high statistical power and resulting high sensitivity of the Shapiro-Wilk test in larger samples (N>50) to even the slightest deviations from normality, the graphical results were considered sufficient to justify the use of linear regression [19-22]. The nonsignificant Breusch-Pagan test (*P*=.70) indicated that homoscedasticity was present [23]. All predictors were correlated with each other in pairs to test for multicollinearity. The highest correlation (0.77) was observed between the *number of sessions* and the *number of completed worksheets*. This indicated that multicollinearity was not present, as there is an assumption that correlation values above 0.8 are a sign of multicollinearity [24]. However, the variance inflation factor (VIF) in the full model with the interactions indicates multicollinearity for the predictors’ *format*, *completed worksheets,* and *number of sessions*, as well as the interactions (VIF>10) [25]. Accordingly, we conducted a stepwise hierarchical regression analysis to localize multicollinearity and compare the models. In the first step (model 1), only the covariates (age, sex, amount of guidance, current psychotherapy, and current antidepressant use) were included as predictors in the model. In the second step (model 2), the format was added. In step 3 (model 3), the use parameters were included. Models 1 to 3 indicated no critical multicollinearity (VIF <2.6). In step 4 (model 4), interaction terms between format and use parameter were included. The interactions did not significantly improve the model and led to increased multicollinearity (VIF >10) [25]. Therefore, a regression model without interactions was used (Table 2). The full model including interaction terms is available in Table S2 in Multimedia Appendix 1.

**Table 2.** Summary of the multiple regression model predicting delta Patient Health Questionnaire-9 scores (negative values indicate a reduction in symptoms; N=167; *R*^2^^a^=0.26; adjusted *R*^2^=0.21).

	B^b^	*P* value
Constant	3.71	.10
Age (years)	–0.01	.66
Sex (male)	–0.39	.59
Antidepressants (yes)	0.44	.48
Psychotherapy (yes)	0.46	.47
Baseline Patient Health Questionnaire-9	–0.61	<.001^c^
Guidance	0.02	.24
Format (browser)	–1.48	.14
Worksheets completed	–0.05	.43
Number of sessions	0.04	.78
Workshops completed	0.06	.83

a *R*2: explained variance of the model.

b B: unstandardized coefficients.

c This result is significant (*P*<.001).

Statistical analyses were performed using R (R Foundation for Statistical Computing) statistics [26]. The significance level was set at α=.05.

## Results

### Comparison of Adherence Parameters

We found no significant differences between the iFD tool and the iFD app in terms of the *number of sessions per week* (*t*_67.393_=0.920; *P*=.36; corrected *P*=.36), the *number of workshops completed* (*t*_76.368_=–1.217; *P*=.30; corrected *P*=.36), and the *number of completed worksheets per week* (*t*_74.099_=0.984; *P*=.33; corrected *P*=.36).

### Comparison of Symptom Reduction

Overall, 37 (n=56, 66.1%) participants from the iFD app pilot study and 130 (n=172, 75.6%) participants from the RCT of the iFD tool completed the PHQ-9 at least once within 5 to 9 weeks. The sociodemographic data of the completers are summarized in Table S1 in Multimedia Appendix 1. Table 3 shows the PHQ-9 scores in both groups before and after the intervention. Table 4 presents a comparison of sociodemographic characteristics between participants who completed at least 1 PHQ-9 assessment within 5 to 9 weeks (completers) and those who did not and were excluded from further analysis (noncompleters).

**Table 3.** Patient Health Questionnaire-9 (PHQ-9) scores at the start of the intervention and after the participants had completed the PHQ-9 at least once within 5 to 9 weeks^a^.

Group	PHQ-9: intervention start, mean (SD)	Last PHQ-9 after 5-9 weeks, mean (SD)	Within-group effect size^b^ (95% CI)
Study iFD app (n=37)	12.62 (4.11)	8.84 (4.51)	0.88 (0.39 to 1.36)
Study iFD tool (n=130)	9.52 (3.37)	6.98 (3.89)	0.70 (0.45 to 0.95)

a The between-group effect size comparing the iFD app and iFD tool groups was 0.27 (95% CI –0.09 to 0.64).

b Effect size is calculated as Cohen *d.*

**Table 4.** Comparison of sociodemographic characteristics between Patient Health Questionnaire-9 (PHQ-9) completers (at least 1 PHQ-9 completion within 5-9 weeks) and noncompleters.

Variables	Completers (n=167)	Noncompleters (n=61)	Comparison, *P* value (BH^a^-adjusted *P* value)
Age (years), mean (SD); median (IQR)	43.45 (12.98); 44.0 (33.5-53.0)	41.18 (12.45); 41 (31.0-50.0)	.23 (.55)
Female, n (%)	126 (75.4)	45 (73.8)	.93 (.93)
Baseline PHQ-9 score, mean (SD)	10.2 (3.76)	10.8 (3.70)^b^	.33 (.55)
Current psychotherapy, n (%)	96 (57.5)	41 (67.2)	.24 (.55)
Current antidepressants, n (%)	102 (61.1)	39 (63.9)	.81 (.93)

a BH: Benjamini-Hochberg.

b n=58.

The multiple regression analysis revealed no significant difference in symptom reduction between the iFD app and the iFD tool (Table 4). The predictors accounted for 25.8% (adjusted *R*^2^=0.21) of the variance in delta PHQ-9. The baseline PHQ-9 score was the only significant predictor of change in delta PHQ-9 (*P*<.001; *b*=–0.61). For a comprehensive overview of the full model results, including all covariates prior to the stepwise regression, see Table S2 in Multimedia Appendix 1.

## Discussion

### Principal Findings

In this study, we compared adherence based on various use parameters between the app version and the browser-based version of an iCBT for the treatment of depression. Although the app version was expected to offer improved accessibility through enhanced smartphone usability and therefore an improved possibility of using the program everywhere and “on the go,” no significant differences in use parameters were observed. Watts et al [14] similarly reported no significant differences in the *number of sessions* between the app and browser versions. Although our study included a broader set of use parameters to gain a more comprehensive insight into user engagement and activity, we arrived at comparable findings. This study also found no significant differences in symptom reduction between the iFD app and the iFD tool, which is not surprising given the similar adherence. Although in a survey many participants stated that they value health apps on their smartphone because they provide a quick and efficient connection to a wealth of information “on the go” [6], this does not seem to contribute to their being used more actively and more frequently than browser-based programs. It is possible that the iFD tool was frequently accessed via a smartphone, which allowed the use of the program “on the go” too, despite the tool’s limited usability on smartphones compared with the iFD app. However, it is notable that in the RCT of the iFD tool, guidance was provided by the study assistants in a standardized manner. In the pilot study of the iFD app, guidance for participants was provided by their practitioners, and the extent of guidance varied considerably; the guidance often being considerably lower than that for the browser-based version, as time is very limited due to a high volume of patients in routine care, a factor that could contribute to reduced adherence [27,28]. In the pilot study, participants reported spending an average of 13.8 minutes over 8 weeks discussing the app with their practitioner. In contrast, with the iFD tool, study assistants spent an average of 29.76 minutes speaking with the participants about the tool over the course of 6 weeks. Therefore, achieving a similar level of adherence nonetheless could still be an indicator of good usability of the iFD app.

### Limitations

The results from this study should be interpreted with caution as they are based on convenience samples with differing recruitment strategies, and the sample size of the pilot study was limited. Additionally, several limitations should be considered. The amount of guidance was included as a control variable; however, in the iFD app study, it was only estimated by participants, whereas in the iFD tool study, it was objectively measured. This limits comparability but still allows for controlling for broad differences. Furthermore, the PHQ-9 instructions differed between the studies: the iFD app participants were asked to rate their symptoms for the past 2 weeks (standard instruction), while participants in the iFD tool study were asked to rate symptoms since their last PHQ-9 completion. In addition, the period between completion of the baseline PHQ-9 and the last PHQ-9 varied between weeks 5 and 9, as the last completed PHQ-9 was included in the analysis and the study duration differed between the studies (iFD app: 8 weeks; iFD tool: 6 weeks). This combination of different instructions and varying periods may limit the comparability of the PHQ-9 changes between the studies. Although no significant differences in symptom reduction were found, these methodological differences remain a limitation. In addition, the iFD tool offers the option of working with self-printed worksheets that could not be measured in the study, which means that the comparability of the *number of worksheets* could also be limited if this option was used frequently. Furthermore, it was not feasible to monitor whether the iFD tool was used on mobile phones—a possibility that exists but does not offer as good a usability experience compared with the iFD app. Additionally, the 2 studies were conducted 4 years apart (iFD tool: 2016‐2018; iFD app: 2022‐2023), during which digital health use and awareness increased [29]. Legislative changes (eg, the Digitale-Versorgungs-Gesetz in 2019, which enables practitioners to prescribe digital interventions as part of routine medical care [30]) and the COVID-19 pandemic (in-person appointments were often not possible) may have contributed to a greater acceptance and use of digital interventions during the period in which the pilot study was conducted.

### Conclusions

This study provides initial evidence that adherence to iCBT content is comparable for browser- and app-based interventions and that symptom reduction is similar for both formats. Despite the limitations of this secondary analysis, it nonetheless offers a rare opportunity to directly compare the same intervention delivered via a mobile app and a browser-based format. The superiority of apps in terms of adherence seems plausible, but this could not be confirmed in this study. Future studies are needed to establish an evidence base for selecting the optimal format.

## Supplementary material

10.2196/58835 Multimedia Appendix 1 Sociodemographic characteristics of Patient Health Questionnaire-9 completers and full regression model–inclusive interaction terms.

## Abbreviations

iCBT: internet-based cognitive behavioral therapy
iFD: iFightDepression
PHQ-9: Patient Health Questionaire-9
RCT: randomized controlled trial
TAU: treatment as usual
VIF: variance inflation factor
