# Supplementary material for: Comparison Between Browser- and App-Based Versions of a Program for Self-Management of Mild to Moderate Depression: Log Data Analysis of a Convenience Sample
Source: JMIR Mhealth Uhealth. 2026 Mar 20;14:e58835. doi: 10.2196/58835 (PMC13004590; doi:10.2196/58835)
Supplement: Multimedia Appendix 1 [file mhealth-v14-e58835-s001.docx]

## Supplementary Material

Table 1. Overview of sociodemographic characteristics of participants who completed the PHQ-9 questionnaire at least once within 5-9 weeks

| Variables | Study iFD-app  (n=37) | Study iFD-tool  (n=130) | Comparison, *P* (BH adjusted *P*) |
| --- | --- | --- | --- |
|  |  |  |  |
| Age in years, mean; (SD); median | 42.84; (14.06); 44.0 | 43.62; (12.71); 44.5 | .761 (.761) |
| Female, n (%) | 21 (56.76) | 105 (80.77) | **.005 (.011)*** |
| Baseline PHQ-9, mean (SD) | 12.62 (4.11) | 9.52 (3.37) | **<.001 (.001) ***** |
| Current psychotherapy, n (%) | 23 (62.16) | 73 (56.15) | .643 (.761) |
| Current antidepressants, n (%) | 15 (40.54) | 87 (66.92) | **.007 (.011) *** |

Abbreviations. PHQ-9: patient health questionnaire, SD: standard deviation, BH: Benjamini-Hochberg method

Table 2: Summary of full multiple regression model including interaction terms predicting delta PHQ-9 (negative values indicate a reduction in symptoms) (N = 167, R^2^ = 0.27, adjusted R^2^ = 0.21)

|  | B | *p* |
| --- | --- | --- |
| Constant | -1.31 | .736 |
| Age | -0.01 | .777 |
| Gender (male) | -0.34 | .642 |
| Antidepressants (yes) | 0.34 | .599 |
| Psychotherapy (yes) | 0.47 | .469 |
| Format (Browser) | 4.07 | .263 |
| Baseline PHQ-9 | -0.58 | <.001*** |
| Guidance | 0.02 | .280 |
| Workshops completed  Worksheets filled in per week  Number of sessions per week | 0.97  -0.01  -0.06 | .115  .929  .852 |
| Format * Workshops completed | -1.12 | .101 |
| Format* Worksheets filled in per week | -0.03 | .825 |
| Format* Number of sessions per week | 0.13 | .718 |

*Notes*. *R^2^*= *explained variance of the model*; B = *unstandardized coefficients*; *p* = p-Value *** result is significant (*p* < .001)
